# Association of tumour necrosis factor-α (*TNF-α*) gene polymorphisms (-308 G>A and -238 G>A) and the risk of severe dengue: A meta-analysis and trial sequential analysis

**DOI:** 10.1371/journal.pone.0205413

**Published:** 2018-10-09

**Authors:** Cho Naing, Norah Htet Htet, Wong Siew Tung, Arun Kumar Basavaraj, Joon Wah Mak

**Affiliations:** 1 Institute for Research, Development and Innovation (IRDI), International Medical University, Kuala Lumpur, Malaysia; 2 Division of Tropical Health and Medicine, James Cook University, Townsville, Australia; 3 School of Medicine, International Medical University, Kuala Lumpur, Malaysia; Academia Sinica, TAIWAN

## Abstract

Individual studies have assessed the association between *TNF-α*-308G>A and *TNF-α*-238 G>A polymorphisms and severity of dengue infection. However, the results are inconclusive and most studies had small sample sizes. The objective of this study was to summarize the evidence of association between *TNF-α*-308 G>A and *TNF-α*-238 G>A and severity of dengue infection.

This study follows the preferred reporting items for systematic reviews and meta- analyses of genetic association studies, recommended by PLOS One. We calculated pooled odds ratio and its 95% confidence interval (CI) to estimate the association between *TNF-α*-308 G>A or *TNF-α*-238 G>A and the risk of severe dengue infections. To determine the information size required for this meta-analysis study, a trial sequential analysis (TSA) was done. Eight studies (640 cases and 1275 controls), which assessed the association of *TNF-α*-308 G>A or *TNF-α*-238 G>A and the risk of DHF were included. Overall, we found no significant association between *TNF-α*-308 G>A and the DHF risk in the allelic model (OR, 0.91; 95% CI, 0.51–1.63), the recessive model (OR,1.32;95%CI,0.73–2.37), the dominant model (OR,0.93;95%CI:0.59–1.47) or the additive model (OR,1.43,95;95%CI:0.79–2.59). There was also no significant association between *TNF-α*-238 G>A and DHF risk under the allele contrast model (OR:1.51;95%CI:0.88–2.58), the recessive model (OR,1.48,95% CI:0.33–6.58), the dominant model (OR,1.48;95%CI:0.56–3.92), or the additive model (OR:1.5;95%CI:0.34–6.69). On subgroup analysis, neither the Asian population nor the non-Asian population showed significant association between *TNF-α*-308 G>A/*TNF-α*-238 G>A and the DHF risk under any genetic models. Leave-one-out meta-analysis showed stability of the results. TSA plots suggested that the sample size in this meta-analysis study was below the required information size.

The findings suggest an inclusive evidence of the association between *TNF-α*-308/ *TNF-α*-238 G>A and the risk of developing severe dengue infection. Large studies with evidence of Hardy-Weinberg equilibrium, assessing gene-gene interactions are recommended.

## Introduction

Dengue fever is endemic in the tropics and sub-tropics and it is the most common arboviral infection caused by dengue viruses (DENV-1, -2, -3, and -4), which are transmitted primarily by the bite of *Aedes aegypti* and *Ae*. *albopictus* mosquitoesThe clinical manifestations of DENV infection vary from mild (asymptomatic, undifferentiated fever, dengue fever) to the severe form of dengue haemorrhagic fever (DHF). It is estimated that 2.5 billion people worldwide are at risk for DENV infection. About 5% of those at risk will have severe forms such as DHF and dengue shock syndrome (DSS), which are potentially fatal [1,2].

The exact pathogenesis of progression from mild to severe forms of dengue infections is not known. A number of studies have suggested that leakage of plasma, which differentiates DHF from DF is attributed to the direct and indirect effects of cytokines or chemical mediators on the vascular endothelial cells [3–5]. In animal models, TNF-α has been implicated in the process of plasma leakage and shock [6].

There is an array of polymorphisms regulating the *TNF-α* gene, of which the two most frequently investigated are at position -308 [7] and at -238 in the promoter region [8]. Meta-analyses have documented the association between *TNF-α* and the increased risk of colorectal cancer under the homozygote model [9], and for pulmonary tuberculosis [10].

Individual studies assessing the association between the *TNF-α*-308 G>A or *TNF-α*-238 G>A promoter polymorphism and the risk of dengue infections in different populations are available in the literature. However, the results are inconsistent. Most of these studies are based on small sample sizes, with low statistical power. A meta-analysis of available individual genetic association studies is valuable to ascertain whether the polymorphisms of *TNF-α*-308 G>A and *TNF-α*-238 G>A gene affect the risk of severe dengue infections. Published meta-analyses in this field are also available, but there are concerns related to the selection of studies included [11], the SNPs included [12], the accuracy of data extracted [12] and genetic models undertaken [11]. Taken together, the objective of this review was to summarize the evidence of association between *TNF-α*-308 G>A or *TNF-α*-238 G>A and severity of dengue infection.

## Materials and methods

The current study conformed to the checklist for meta-analysis of genetic association studies specified for PLOS One approach [13] (S1 Table).

### Study search

The potentially relevant studies were searched in the health-related databases of PubMed, Ovid Medline, google scholar and web of science and with the combination of the keywords with Boolean operators: “dengue” OR “DHF” OR “dengue haemorrhagic fever” OR “DSS” OR “dengue shock syndrome” AND “tumour necrosis factor- alpha-308” OR “rs1800629” OR “TNF-α-308 G>A” OR “tumour necrosis factor-alpha” OR “*TNF-α*-238 G>A”. The details of search in PubMed database is provided in S2 Table.

The search was restricted to the publications in English until January 2018. We also searched manually the references of articles retrieved and relevant systematic reviews to find any additional studies.

### Inclusion criteria

Human studies of any sample size that assessed severe dengue infection were included, if (1) *TNF-α*-308 G>A (including rs 1800629), *TNF-α*-238 G/A or both were investigated; (2) they were case-control design (retrospective or nested case-control) with an outcome of severe dengue infections (DHF/DSS) measured as incidence or prevalence, and (3) there was sufficient information to extract the genotype frequency both in cases and controls.

Severe dengue infection in the current analysis encompassed DHF and/or DSS as defined in the primary studies. Studies, which did not meet the inclusion criteria were excluded. Studies based on family or sibling-pairs were not considered.

### Data extraction

Two investigators independently screened the titles and abstracts, and then selected the relevant full-text articles, according to the inclusion criteria. The two investigators independently extracted data, using a piloted data extraction form. Information collected from each study included: first author, publication year, country, study setting (e.g. hospital-based or population-based), the frequency of cases and controls, the racial descent (Asian or non-Asian), method of genotyping and genotype/allele frequencies in cases and controls. If allele frequency was zero, then we added 1 to all allele, following the Laplace approximation [14]. Any discrepancy between the two investigators were resolved by consensus.

### Assessment of the methodology quality

The two investigators independently evaluated the methodological quality of studies with the use of the Newcastle-Ottawa Scale (NOS) [15]. These scores are based on three factors such as the selection of the study groups (4 points), the comparability of the groups (2 points) and the ascertainment of the exposure (3 points). The total score for each study ranged between 0 (the worst) and 9 (the best). As described in a published systematic review [16], we adapted the cut-off score points as good (≥7), moderate (≥5) and poor (≤4) for study quality assessment. Any discrepancy between the two investigators was resolved by consensus.

### Statistical analysis

An evidence of HWE in the control population was assessed (if not provided in the study included) or re-assessed (if already provided) in this meta-analysis with the use of the goodness-of-fit test (*p*>0.05) [17].

Following a method described [18], the strength of the association between *TNF-α*-308 G>A or *TNF-α*-238 G>A and the risk of developing DHF/DSS was estimated using odds ratios (ORs) and its 95% confidence intervals (CIs) for individual studies included. Heterogeneity was evaluated with the use of *I*^2^ test. The *I*^2^ test values indicates the percentage of total variation across studies that is attributable to the heterogeneity rather than chance. *I*^2^ values lie between 0% and 100%, with greater than 50% regarded as having substantial heterogeneity [19]. For pooling of the estimates across studies included, the summary ORs and its 95% CIs were calculated with random-effect model (The Der Simonian and Laird method), based on the presence of statistical heterogeneity of the studies. Otherwise, fix-effect model (the Mental-Haenszel method) was used. We calculated the summary ORs and its 95% CIs in four genetic models: the allelic contrast model (A vs G), the dominant model (AA+GA vs GG), the recessive model (AA vs GA+GG), and the additive model (AA vs GG). To investigate the stability of results, a sensitivity analysis was done with leave-one-out meta-analysis by omitting one study at a time [20]. All eight studies included used different case definitions of dengue severity. Hence, a sensitivity analysis was done with the studies that used the WHO 1997 definition for dengue severity. We did not assess the publication bias by visual inspection of funnel plots in the absence of a minimum 10 studies required for this assessment [21,22].

Trial sequential analysis (TSA), a tool for adjustment of random error risk, was done for estimation of the required information size [23]. It is classified as ‘firm evidence of effect’ or ‘potentially spurious evidence of effect’; this classification depends on the cumulative Z-curve that cross the monitoring boundaries or not [24].

Forest plots and leave-one-out meta-analysis were done with RevMan 5.3 (The Cochrane collaboration, Copenhagen) and *meta* package in *R* 3.4.3 software (The *R* Foundation), respectively. TSA plot was done with TSA software (Copenhagen Trial Unit, Centre for Clinical Intervention Research, Copenhagen).

## Results

### Study search results

**Fig 1:**
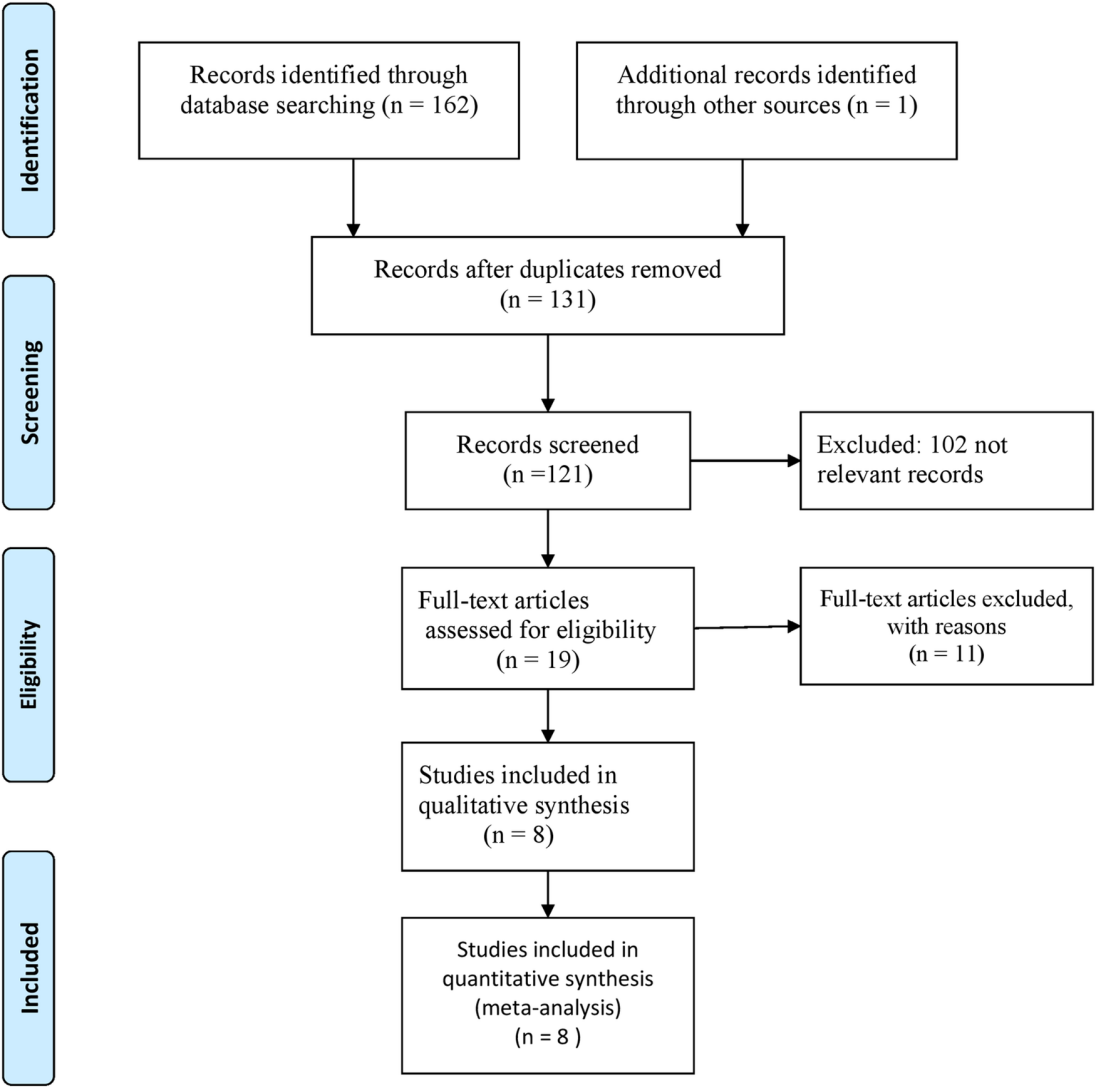
PRISMA flow diagram showing the study selection process.

Fig 1 shows a four-phase study selection process in this meta-analysis. A total of 167 records were found in the initial search, of which 32 were duplicates. After screening of abstracts, 19 full-text articles were retrieved, based on the inclusion criteria. Finally, eight studies (with 640 cases and 1275 controls) were identified for this review [25–32]. All these eight studies assessed *TNF-α*-308 G>A. Only 3 studies assessed both *TNF-α*-308 G>A and *TNF-α* 238 G>A [26,30,32] in the risk of severe dengue. Summary of the 11 excluded studies [33–43] were provided in S3 Table.

### Study characteristics

Table 1 presents the characteristics of the nine included studies, along with their genotype and allele frequency distribution of *TNF-α*-308 G>A and *TNF-α*-238 G>A gene polymorphisms. Two studies each were from Brazil [27, 31] and Mexico [26,32], while one study each from Cuba [25], India [28], Malaysia [30] and Sri Lanka [29]. Study samples varied from 43 cases [25] to 196 [30]. The years of publication spanned from 2010 and 2017. The single nucleotide polymorphisms (SNPs) in two studies deviated from HWE [25, 26]. All these eight studies were with moderate to good methodological quality, based on the NOS criteria (S4 Table).

**Table 1:** Characteristics of the studies included in the meta-analysis.

Study	Yr	Ref #	Country	Setting	Diagnostic criteria	Genotyping	DHF cases/ Healthy controls	Age group	Study quality score	Cases		Controls	
										A/G	AA/GA/GG	A/G	AA/GA/GG
**Perez**	2010	25	Cuba	population based	1997 WHO criteria	PCR-SSP	43/99	adults	7	29/57	9/11/23	21/145	14/7/69
**García-Trejo**	2011	26	Mexico	hospital based;	1997 WHO criteria	PCR-RFLP	45/169	adults	7	0/64	0/0/32	23/315	1/21/147
**Xavier-Carvalho**	2013	27	Brazil	hospital based;	2009 WHO/TDR	Real-time PCR	88/335	children	8	20/144	2/16/64	73/585	6/66/271
**Alagarasu**	2015	28	India	hospital based	1999 WHO criteria	ARMS-PCR	45/108	adults	8	6/84	0/6/39	13/203	0/13/95
**Fernando**	2015	29	Sri Lanka	hospital based	2011 WHO criteria	ARMS-PCR	107/52	adults	5	14/200	2/10/95	28/107	2/13/47
**Sam**	2015	30	Malaysia	hospital based	1997 WHO criteria	PCR-RFLP	196/120	Mostly adults	8	17/373	0/17/178	24/216	1/22/97
**dos Santos**	2017	31	Brazil	hospital based	1997 WHO criteria	Real-time PCR	49/135	adults	7	10/88	0/10/39	39/231	3/33/99
**Sanchez-Leyva**	2017	32	Mexico	hospital based	1997 WHO criteria	PCR-RFLP	67/257	adults	9	12/122	3/6/58	35/497	2/31/224

### Quantitative estimates

**Fig 2:**
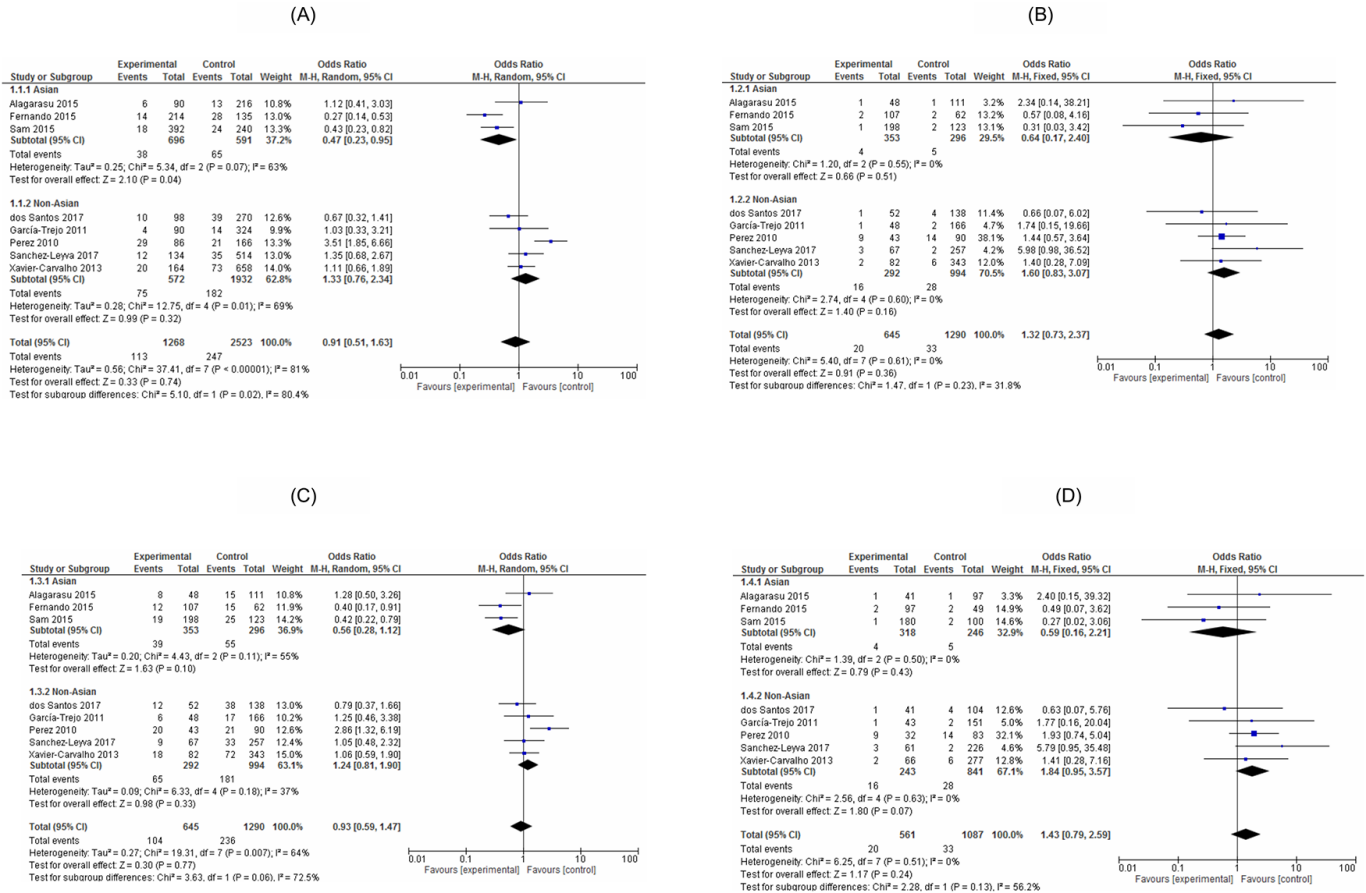
Forest plot of the associated *TNF-α*-308 G>A and dengue haemorrhagic fever (A: Allele contrast model, B: Recessive model, C: Dominant model, D: Additive model).

Overall, there were no significant associations between *TNF-α*-308 G>A and the DHF risk under the allelic model (OR,0.91;95% CI,0.51–1.63,*I*^2^:81%), the recessive model (OR,1.32, 95%CI; 0.73–2.37,*I*^2^:0%), the dominant model (OR,0.93;95%CI:0.59–1.47,*I*^2^:64%) and the additive model (OR,1.43;95%CI:0.79–2.59, *I*^2^:0%). On stratification, the Asian population or the non-Asian population showed no significant relationships between *TNF-α*-308 G>A and the DHF risk under any genetic models, except a recessive model with the Asian population (0.47, 95%CI: 0.23–0.85, *I*^2^: 67%) (Fig 2A, 2B, 2C and 2D).

**Fig 3:**
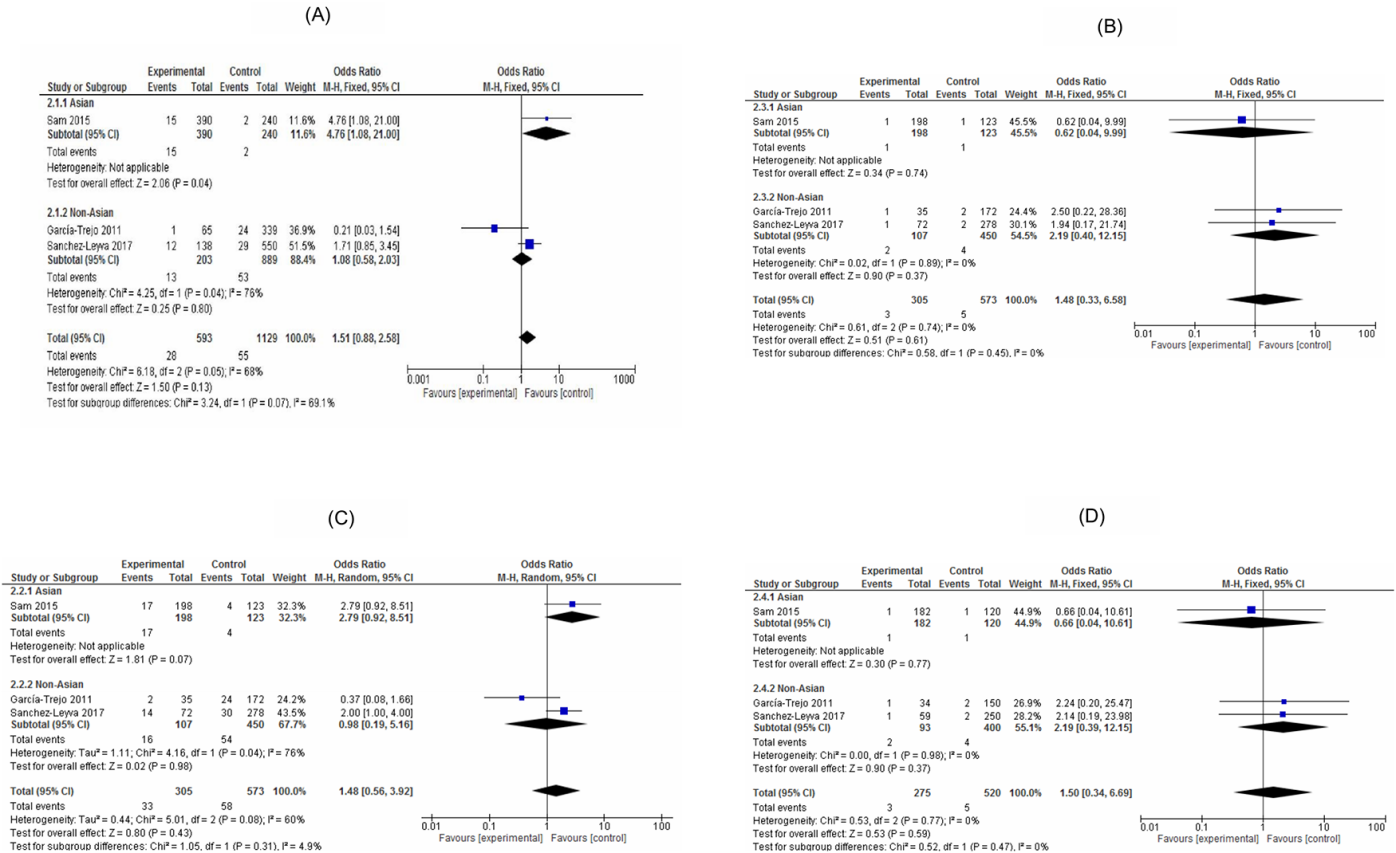
Forest plot of the associated *TNF-α*-238 G>A with dengue haemorrhagic fever (A: Allele contrast model; B: Recessive model, C: Dominant model, D: Additive model).

For *TNF-α*-238 G>A, overall there was no significant association with the DHF risk in the allelic model (OR,1.51;95%CI,0.88–2.58,*I*^2^:68%), the recessive model (OR,1.48,95%CI,0.33–6.58,*I*^2^:0%), the dominant model (OR,1.48;95%CI:0.56–3.92,*I*^2^:60%) and the additive model (OR,1.5;95%CI:0.34–6.69,*I*^2^:0%). Also, a stratified analysis with the Asian population or the non-Asian population did not show significant association between *TNF-α*-238 G>A polymorphism and the risk of DHF in any genetic models (Fig 3A, 3B, 3C and 3D).

**Fig 4:**
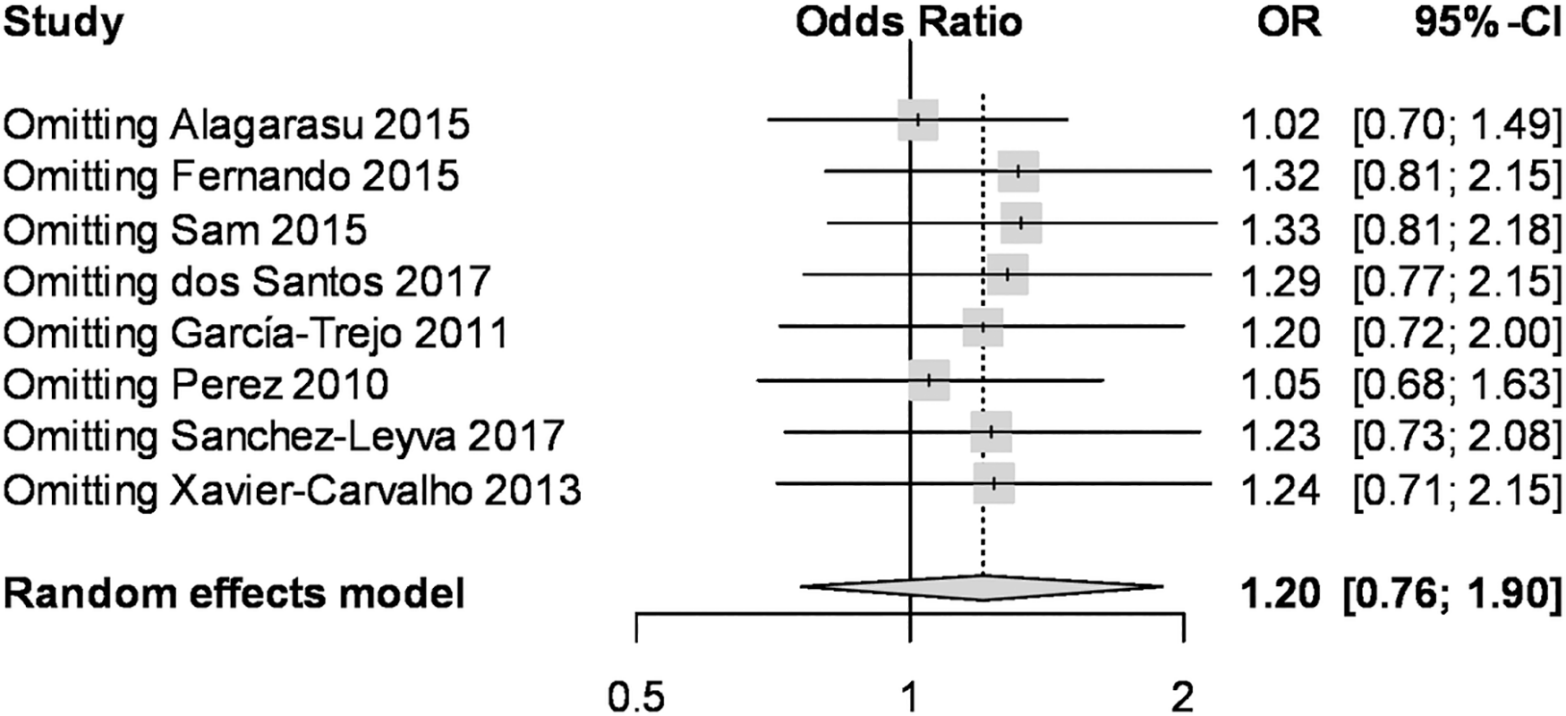
Leave-one-out meta-analysis for the *TNF-α*-308 G>A under the dominant model.

To evaluate the impact of individual data on the pooled ORs, sensitivity analysis was performed using leave-one-out meta-analysis. For instance, the overall estimate of dominant model of *TNF-α*-238 G>A remained as having no statistical significance, on omitting any single study (Fig 4), indicating that the results are stable. Another sensitivity analysis was done with the studies that used the WHO 1997 definition for dengue sensitivity. We also found no significant association between *TNF-α*-308 G>A polymorphism and the risk of DHF in allelic genetic models (OR: 1.08, 95%CI: 0.49–2.37, I2: 83%) (S5 Table). This was also true for the other genetic models.

### Trial sequential monitoring

**Fig 5:**
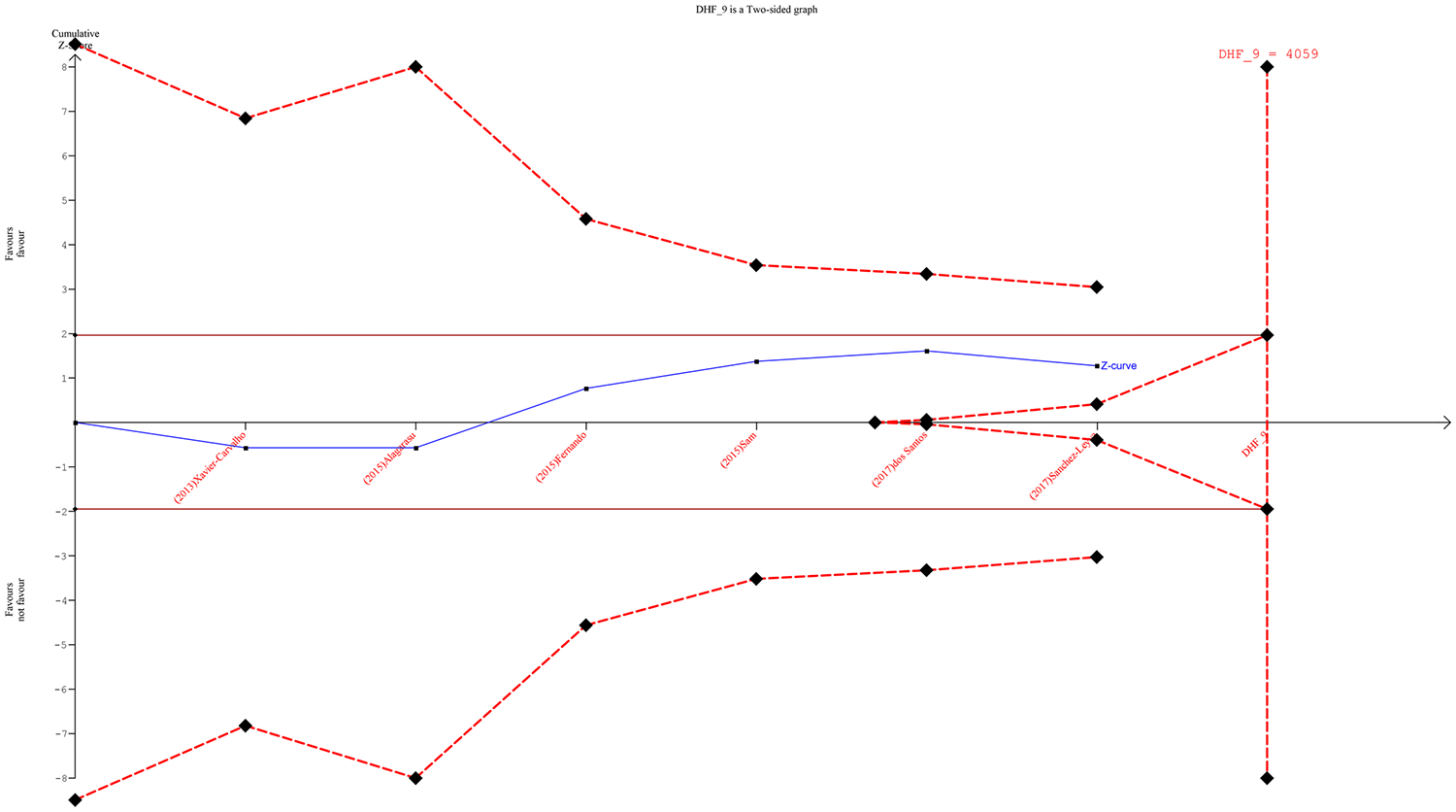
Trial sequential monitoring plot of TNF-α-308 G>A in severe dengue infection.

We performed TSA with six studies that followed evidence of HWE [27–32]. We plotted with the use of an overall type I error of 5% and type II error of 20%. The currently included total participants in this meta-analysis was below the required information size of 4059. As the cumulative Z-curve (blue full line with quadratic indications of each trial) touches the boundary for futility, it will be unlikely to reach a statistical significance *p* <0.05, even the required information size of 4059 is obtained (Fig 5). This was also true for *TNF- α* -238 G>A.

### Discussion and conclusions

The current study provides evidence on the relationship between *TNF-α*-308 G>A or *TNF-α*-238 G>A and the risk of DHF, and the major observations are as follows;

1. In a combined population as well as subgroups of Asian and non-Asian groups, both SNPs (*TNF-α*-308 G>A and *TNF-α*-238 G>A were not significantly associated with the DHF risk in almost all genetic models investigated.
2. The TSA plot revealed that the required information size for evidence of effect was insufficient to draw a firm conclusion.

The plasma leakage in DHF occurs up to several days after viremia has been reduced or disappeared, suggesting the role of an immune-mediated mechanism in DHF [6, 7]. *TNF-α* gene was first cloned in 1985 and it is expressed in diverse cells including macrophages and tumour cells. Although an exact mechanism is not fully understood, the action of *TNF-α* includes stimulating the acute phase response that lead to increase in the C-reactive protein levels, and producing IL-1 oxidant and inflammatory lipid prostaglandin E-2 [7]. An *in vitro* study has reported that acute dengue sera had significantly high TNF-α levels, and the endothelial activation was inhibited more than 70% with pre-treatment of monoclonal antibodies against *TNF-α*. This could explain why the transient plasma leakage occurred mainly at serosal tissues in dengue cases [4,44].

A published meta-analysis has documented that *TNF- α* -308 G>G genotype and allele G confer susceptibility to symptomatic dengue, while *TNF- α* -308 G>A genotype and allele A confer protection [12]. The current analysis, which focussed on the severe form of infection with potential fatality such as DHF/DSS, showed no significant relationship between *TNF-α*-308 G>A and the DHF risk. This difference is related to variation in immunopathology of the target conditions as the clinical outcome of symptomatic dengue and severe dengue, are not at the same degree of importance. The findings in this analysis were comparable with another meta-analysis of seven individual studies (n = 1533), suggesting that *TNF-α*-308 G>A was not associated with DHF [12]. Although there are more participants in this analysis the results retained the evidence of no association. Moreover, the current review assessed more than one SNP.

The lack of association either protective or enhancement of the DHF risk in the current analysis might be due to insufficient number of participants, which is supported by the TSA plot. It showed that the included eight studies in this meta-analysis could only contribute to 65.7% of the required information size. This implied there was insufficient information to provide conclusive results.

Another possible reason of a lack of association in the present analysis may be due to the limited role of *TNF-α* in the development of DHF risk. In animal models, *IFN-γ* does not directly induce plasma leakage, but promotes *TNF-α* production through activated monocytes [45] and interactions with TNF-α to activate endothelial cells [46]. As such, it is likely that the combined cytokines including *TNF-α*-308 G>A and other cytokines are important to establish the significant role in the DHF risk. This was supported by a study in Malaysia that showed a significant correlation between interactions of *TNF-α*-308G>A genotype and *IL10* non-GCC haplotypes, *IL12B* pro homozygotes (pro1/pro1, pro2/pro2) and *IL-12B* 3’UTR AC and the protective effects against DHF/DSS [30]. It has been suggested that the interactions of *TNF-α*, *IFN- γ* and activated complement proteins enhance plasma leakage of endothelial cells in secondary dengue infection [42]. A small number of studies did not allow to perform pooled analysis with these combined cytokines. The studies identified for the current meta-analysis used different case definition of dengue severity. Some used 2009 WHO definition, some used 1997 definition. Both definitions of the WHO 1997 and 2009 may include the patients with different disease severity, which may not relate to vascular permeability. However, a sensitivity analysis solely on the five studies that used WHO 1997 definition remained the non-significant association between *TNF-α*-308 G>A and the risk of severe dengue. This implied that the criteria for dengue severity had no impact on the direction of association.

### Study limitations

We acknowledge the limitations of the present review. Approximately 75% of the global population residing in the Asia Pacific Region are exposed to DENV [47]. Only 36% of the included studies were carried out in the Asia Pacific Region; there is a likely selection bias with geographical imbalance. The current analysis was done with the unadjusted raw data provided in the primary studies, whereas most of the estimations in the individual studies might have been adjusted with common factors (i.e. age, gender). Hence, the pooled ORs in this meta-analysis may be slightly differed from what was reported in the primary adjusted studies. If cases and controls have been genotyped in separate batches in the primary studies, a bias related to differential misclassification of exposure is a concern. Due to the small sample sizes in most of the included studies, type II errors had limited to find the significant differences between the cases and controls or amongst the ethnic groups. However, meta-analysis is a retrospective pooling of published studies, and type II errors are less likely than in individual studies [48]. We planned to do stratified analysis by age group. This was not possible in view of only one study with children. Moreover, there might be some extent of interaction with *TNF-α*-238 G>A or *TNF-α*-308 G>A and other genes (gen-gen interaction/synergism) or other potential confounding factors such as nutritional status of the patients included [16]. Hence, findings in this meta-analysis should be interpreted with cautions.

There are some strengths in this meta-analysis compared with other reviews in this field. To be comprehensive, we have added more than one SNP in the present analysis. We performed this review, following the guideline for meta-analysis of genetic association studies [13]. Moreover, the TSA technique was useful to adjust random-error risk. An add-on TSA approach to this field will highlight to researchers about the optimal sample size to adopt. This will help the researchers and policy makers to reach a more balanced conclusion on the estimates [24], which is the case in this study. In summary, the findings suggest that it is inconclusive to determine whether any of these two SNPs is associated with the risk of severe dengue infection. Additional case-control studies on these two SNP may likely to change the estimates. Large studies with evidence of HWE, assessing these SNPs and other SNPs as gene-gene interactions are recommended.

## Supporting information

S1 Table Checklist for meta-analysis of genetic association studies—PLos One approach. (DOC)

S2 Table Search strategy in PubMed. (DOC)

S3 Table The excluded studies and reasons for exclusion. (DOC)

S4 Table The methodological quality of the included studies. (DOC)

S5 Table Sensitivity analysis of the allelic model using the WHO 1997 criteria. (DOC)
