# Supplementary material for: Association of tumour necrosis factor-α (TNF-α) gene polymorphisms (-308 G>A and -238 G>A) and the risk of severe dengue: A meta-analysis and trial sequential analysis
Source: PLoS One. 2018 Oct 9;13(10):e0205413. doi: 10.1371/journal.pone.0205413 (PMC6177181; doi:10.1371/journal.pone.0205413)
Supplement: S1 Table — (DOC) [file pone.0205413.s001.doc]

**S1 Table**

**Checklist for meta-analysis of genetic association studies—PLOS ONE approach**

|  | Item | Section name and paragraph number within manuscript |
| --- | --- | --- |
|  | **Introduction** |  |
| 1 | Provide a detailed justification for the polymorphism studied; if a single polymorphism was analyzed, give details as to why others were not included in the meta-analysis. | # 93-102 |
| 2 | Provide a detailed justification for the population(s) and clinical condition studied. | # 84-90 |
|  | **Methods** |  |
| 3 | Provide full details of the search strategy employed; outline the full electronic search strategy –specific combination of keywords and any limits applied- for at least one database. Specify whether synonyms of polymorphisms/genes (e.g. SNP number) were searched. | Study Search  S2 Table |
| 4 | Report full details on the inclusion and exclusion criteria applied for selecting studies. Please list the excluded articles and the reasons for exclusion of each article in a supplementary file. | Inclusion criteria  #127- 134  Exclusion criteria  S3 Table |
| 5 | Provide details on how the quality of the studies included in the analyses was assessed. | Assessment  Methodology quality  # 146-153  S4 Table |
| 6 | Describe steps taken to contact study authors to identify additional studies and to request missing data. | Complete data are available from the papers. |
| 7 | Describe how environmental effects were adjusted for, if this adjustment was not conducted, outline the reasons for this. | Not included in the primary studies. |
| 8 | Describe the methods of handling heterogeneity/between-study variance. | Statistical analysis  #156-176 |
| 9 | Describe how the Hardy-Weinberg equilibrium and linkage disequilibrium were assessed. | Statistical analysis  #156-158 |
| 10 | Describe and justify the choice of model for the analyses (per-allele vs per-genotype vs genetic model-free, random effects vs fixed effects). | Statistical analysis  #159-176 |
| 11 | Describe whether a sensitivity analysis has been completed. | Statistical analysis  #173-176 |
| 12 | Describe whether an assessment of the effects of population stratification has been conducted. |
| 13 | Describe whether study-specific results have been assessed and if so the reasons for this (e.g. forest plot) | Figure 2- 3 |
|  | **Results** |  |
| 14 | Include flow diagram for the studies included in the meta-analysis as the first figure for the manuscript. | # 188  Figure 1 |
| 15 | Report details on allele/genotype prevalence. | Table 1  # 218-219 |
| 16 | Report the effect size estimates and p values for each analysis. | Quantitative estimates  # 209-242  Figure 2-3 |
|  | **Discussion** |  |
| 17 | Discuss the limitations of the meta-analysis, including genotyping errors/bias and publication bias. | Study Limitations  # 313-329 |
| 18 | If the meta-analysis identifies an association within a subgroup of the population studied but not another, discuss the implications of these results, and if applicable the possibility of subgroup-specific publication bias. | Whole population of the study were assessed. |
| 19 | Discuss the suitability of the sample size employed to the research question and the power of the study. | # 330-340 |
