# Supplementary material for: Association of tumour necrosis factor-α (TNF-α) gene polymorphisms (-308 G>A and -238 G>A) and the risk of severe dengue: A meta-analysis and trial sequential analysis
Source: PLoS One. 2018 Oct 9;13(10):e0205413. doi: 10.1371/journal.pone.0205413 (PMC6177181; doi:10.1371/journal.pone.0205413)
Supplement: S2 Table — (DOC) [file pone.0205413.s002.doc]

**S2 Table. Search strategy in PubMed**

((((("tumour necrosis factor"[All Fields] OR "tumor necrosis factor-alpha"[MeSH Terms] OR ("tumor"[All Fields] AND "necrosis"[All Fields] AND "factor-alpha"[All Fields]) OR "tumor necrosis factor-alpha"[All Fields] OR ("tumor"[All Fields] AND "necrosis"[All Fields] AND "factor"[All Fields]) OR "tumor necrosis factor"[All Fields]) OR (TNF[All Fields] AND 306[All Fields])) OR (TNF[All Fields] AND 238[All Fields])) AND ("dengue"[MeSH Terms] OR "dengue"[All Fields])) AND "dengue"[MeSH Terms]) AND ("case-control studies"[MeSH Terms] OR ("case-control"[All Fields] AND "studies"[All Fields]) OR "case-control studies"[All Fields] OR ("case"[All Fields] AND "control"[All Fields]) OR "case control"[All Fields])
