# Supplementary material for: Association of tumour necrosis factor-α (TNF-α) gene polymorphisms (-308 G>A and -238 G>A) and the risk of severe dengue: A meta-analysis and trial sequential analysis
Source: PLoS One. 2018 Oct 9;13(10):e0205413. doi: 10.1371/journal.pone.0205413 (PMC6177181; doi:10.1371/journal.pone.0205413)
Supplement: S3 Table — (DOC) [file pone.0205413.s003.doc]

S3 Table. Excluded studies and reasons for exclusion

| Study | Reference no. | Main reason |
| --- | --- | --- |
| Vitarna, 1991 | 33 | No genotype frequency |
| Fernandez-Mestre,2004 | 34 | No healthy controls |
| Lei Huan-Yao,2008 | 35 | No primary data |
| Restrepo, 2008 | 36 | No genotype frequency |
| Nascimento,2009 | 37 | No genotype frequency |
| Vejbaesyam, 2009 | 38 | Unable to extract allele frequency |
| Alagarasu, 2013 | 39 | The same patients of an included study [26] |
| Chuansumrit, 2013 | 40 | No genotype frequency for DHF |
| Mathew,2014 | 41 | No genotype frequency |
| Dettogni,2015 | 42 | No controls |
| Feitosa,2016 | 43 | No genotype frequency |
