# Supplementary material for: Association of tumour necrosis factor-α (TNF-α) gene polymorphisms (-308 G>A and -238 G>A) and the risk of severe dengue: A meta-analysis and trial sequential analysis
Source: PLoS One. 2018 Oct 9;13(10):e0205413. doi: 10.1371/journal.pone.0205413 (PMC6177181; doi:10.1371/journal.pone.0205413)
Supplement: S4 Table — (DOC) [file pone.0205413.s004.doc]

**S4 Table** The methodological quality of the included studies

| 1^st^ Author | Year of publication | Ref # | Selection | | | | Compar-ability | Outcome | | | | Total Stars |
| --- | --- | --- | --- | --- | --- | --- | --- | --- | --- | --- | --- | --- |
| (a) |  |  | (b) representativeness | (c ) selection of non-exposed cohort | (d) ascertainment of exposure | (e ) outcome of interested is not at the start of study comparability | (f) comparability | (g) assessment of outcome (record linkage) | ( h) assessment of outcome  (independent or blind assessment) | (i) was follow-up long enough for outcomes to occur | (j) adequacy of follow up of cohorts | (h) |
| Perez | 2010 | 25 | * | * | * | * | * | * |  | * | * | 8 |
| García-Trejo | 2011 | 26 | * | * |  |  | * |  | * | * |  | 6 |
| Xavier-Carvalho | 2013 | 27 | * | * | * |  | * | * | * | * |  | 7 |
| Alagarasu | 2015 | 28 | * | * | * | * | * |  | * |  |  | 6 |
| Fernando | 2015 | 29 | * | * | * |  | * |  | * |  |  | 5 |
| Sam | 2015 | 30 | * | * |  | * | * | * | * |  |  | 6 |
| dos Santos | 2017 | 31 | * | * | * | * | * |  | * |  |  | 6 |
| Sanchez-Leyva | 2017 | 32 | * | * | * | * | * |  | * | * |  | 7 |
