# Supplementary material for: Association of tumour necrosis factor-α (TNF-α) gene polymorphisms (-308 G>A and -238 G>A) and the risk of severe dengue: A meta-analysis and trial sequential analysis
Source: PLoS One. 2018 Oct 9;13(10):e0205413. doi: 10.1371/journal.pone.0205413 (PMC6177181; doi:10.1371/journal.pone.0205413)
Supplement: S5 Table — (DOC) [file pone.0205413.s005.doc]

S5 Table. Sensitivity analysis of the allelic model using the WHO 1997 criteria

| Study  [reference No] | Cases | Controls | Odds ratio  [95% confidence interval] |
| --- | --- | --- | --- |
| Perez 2010 [25] | 29/86 | 21/166 | 3.51  [1.85, 6.66] |
| García-Trejo 2011 [26] | 4/90 | 14/324 | 1.03  [0.33, 3.21] |
| Sam 2015 [30] | 18/392 | 24/240 | 0.43  [0.23, 0.82] |
| dos Santos 2017 [ 31] | 10/98 | 39/270 | 0.67  [0.32, 1.41] |
| Sanchez-Leyva 2017 [32] | 12/134 | 35/514 | 1.35  [0.68, 2.67] |
| Total |  |  | 1.08  [0.49, 2.37] |
